# A case report of children's divergent dislocation of the elbow and review of literature

**DOI:** 10.1097/MD.0000000000004772

**Published:** 2016-11-04

**Authors:** Yongtao Wu, Hai Jiang, Wusheng Miao

**Affiliations:** Department of Pediatric Orthopedics, Hong Hui Hospital, Xi’an Jiao Tong University College of Medicine, Shaanxi, China..

**Keywords:** divergent dislocation of the elbow, literature, review

## Abstract

**Background:**

The divergent dislocation of the elbow is not common in children, and the imaging is difficult and challenging. This often leads to misdiagnosis or inappropriate treatment. The literature has reported a total of 19 cases currently.

**Methods:**

A 10-year-old girl with divergent dislocation of the elbow was admitted in our department in November 2013. When playing basketball, her right elbow was injured on the concrete floor. After injury, her right elbow joint became severely swollen, with obvious deformity. The anteroposterior X-ray of elbow showed right olecranon and coronoid fractures, the proximal radioulnar separation, and displacement; the lateral X-ray showed the posterior dislocation of right elbow.

**Results:**

Under local anesthesia, right elbow manual reduction was performed, and after reduction, 3-dimensional computed tomography reconstruction displayed good reduction of the elbow dislocation. The fracture of coronoid displaced minimally, but the olecranon fracture showed great displacement which underwent the open reduction and internal fixation. Postoperatively, a plaster splint was applied for protection, with regular outpatient follow-ups. At the end of the normal follow-up, the active ROM of the right elbow joint was 5° to 130° and with normal rotation.

**Conclusion:**

Therefore, through the treatment of this case and the literature review, we believe that for children, most divergent dislocations of the elbow may achieve a better clinical result with closed reduction, and we also believe that after surgery or closed reduction, in the follow-up, proper function exercise is an important condition for the rehabilitation of children. For such patients, correct diagnosis and timely treatment can help to avoid joint dysfunction of elbow.

## 1 The introduction of divergent dislocation of the elbow

Dislocations of the elbow joint are not common in children, accounting for about 6% of the elbow fractures and dislocations; based on the relative position of the proximal radioulnar joint to the distal humerus, it can be divided into posterior, anterior, medial, and lateral dislocation, among which posterior dislocation is most common; part of elbow dislocations can be combined with ulnar, radial, and humerus fractures, and so on.^[1]^ The divergent dislocation of the elbow is rarely seen as a clinical subtype of posterior dislocation of the elbow and the imaging is difficult and challenging. This often leads to misdiagnosis or inappropriate treatment. The literature has reported a total of 19 cases currently. A child with divergent dislocation of the elbow was admitted in our department in November 2013, and the case is reported and the literature of such disease is reviewed here in order to study the clinical features, diagnosis, and treatment of the fracture and dislocation to avoid misdiagnosis. The report goes as follows.

## 2 Case report

**Figure 1:**
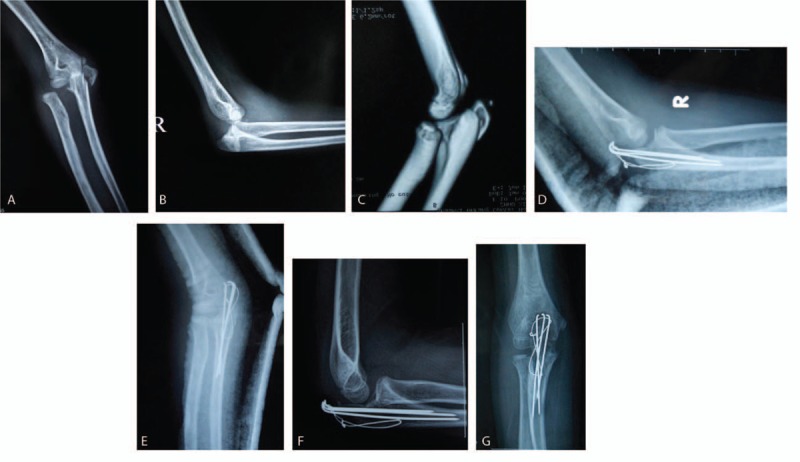
10-year-old girl, preoperative anteroposterior film showed right olecranon and coronoid fractures, and the proximal radioulnar separation and displacement (A), lateral radiographs showed right elbow posterior dislocation (B); after close reduction, CT showed the correction of right elbow dislocation, the good reduction of coronoid fracture, and a large displacement of olecranon fracture (C). Postoperative X-ray showed the good reduction of fracture-dislocation and reliable fixation (D, E); 1 year after surgery, x-ray showed that right lateral elbow dislocation was well reset, and fracture healed (F, G). CT = computed tomography.

A 10-year-old girl accidentally fell when playing basketball and her right elbow was injured on the concrete floor. After injury, her right elbow joint became severely swollen, with obvious deformity at right elbow. Examinations showed no obvious injury symptoms in the radial nerve, ulnar nerve, median nerve, and brachial artery. Immediately after injury, the anteroposterior (AP) x-ray of elbow showed right olecranon and coronoid fractures, the proximal radioulnar separation, and displacement; the lateral x-ray showed the posterior dislocation of right elbow. Under local anesthesia, right elbow closed reduction (CR) was performed, and after CR, 3-dimensional computed tomography reconstruction displayed good reduction of the elbow dislocation. The fracture of coronoid displaced minimally, but the olecranon fracture showed great displacement with an intra-articular fracture. After perioperative preparation, the right olecranon fracture underwent the open reduction and internal fixation. During the surgery, mobilizing the elbow joint showed the stability of the anterior, posterior, and lateral side, so ligaments and capsule around the elbow were not explored. The surgery ended; postoperatively, a plaster splint was applied for protection, with regular outpatient follow-ups (Fig. 1). After the approval of the institutional review board of our hospital, we reviewed the clinical and radiologic outcomes of patient retrospectively.

## 3 Discussion

The divergent dislocation of the elbow is a complex fracture and dislocation of the elbow, rarely seen in clinic, so the initial diagnosis is often difficult to confirm; and is easily misdiagnosed as posterior Monteggia fracture-dislocation, or terrible triad of the elbow. A delay in diagnosis was predictive of noticeable losses in range of motion. Subsequent open reduction (OR) in a delayed setting can result in decreased range of motion and elbow dysfunction which resulting in serious complications affecting children's living and learning. Thus early diagnosis and reasonable treatment of the divergent dislocation of the elbow are of great importance for the patient. Warmont^[2]^ first described the disease in 1854, with neither radiographs nor follow-up examination, and the earliest radiologic literature data were what was reported by DeLee^[3]^ in 1981, and later, 18 cases were reported in the literature one after another (Table 1).

**Table 1:**
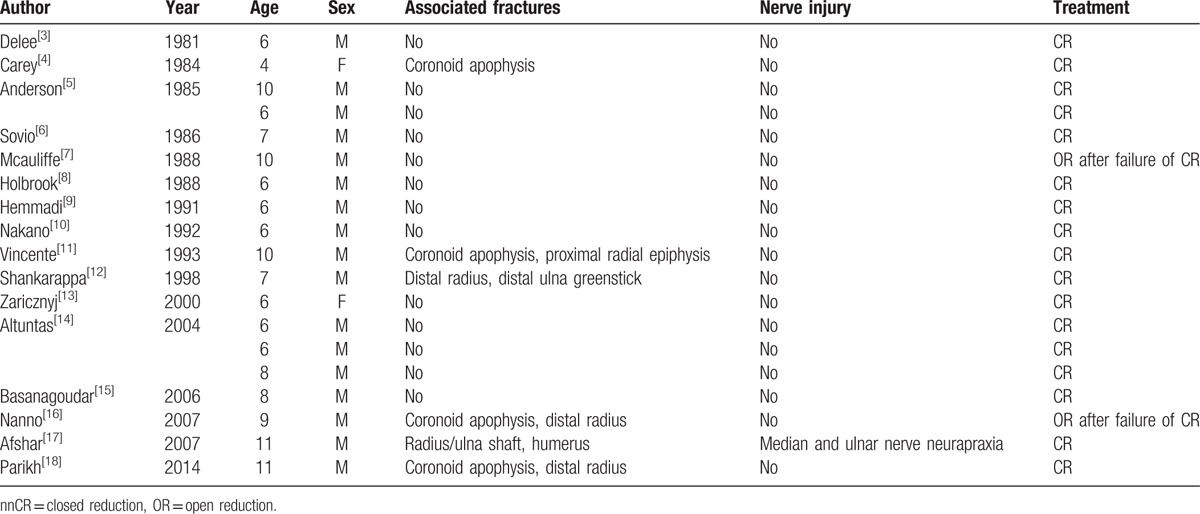
Divergent elbow dislocations in the pediatric population.

Divergent dislocation of the elbow is clinically divided into 2 types, namely, transverse divergent dislocation and AP divergent dislocation.^[13]^ The transverse divergent dislocation is more common in clinic. There is no consensus among many researchers about the mechanism of divergent dislocation of the elbow. As reported by Sovio and Tredwell,^[6]^ the divergent dislocation of the elbow was arisen as a consequence of fall on the outstretched hand with the body pivoting on the hand, causing a hyperpronation force imposed on the elbow. According to Zaricznyj,^[13]^ however, the extended or hyperextended elbowing valgus was observed in the patient, also with the forearm almost supinated. Such injury is commonly explained by an axial force imposed on the outstretched hand with slight flexion combined with pronation due to injury, consequently the distal humerus being presented as a wedge driven between the proximal radius and ulna.^[9]^ The pathologic anatomic features mainly involve the dislocation of three joints including the humeroulnar, radiocapitular, and radioulnar. It also combined with severe soft tissue injuries around the elbow, including the anterior capsule, the collateral ligaments, the radioulnar or annular ligaments, and the interosseous membrane of the radius and ulna. In some cases, avulsion fracture of the elbow may also occur. However, children have less injury in soft tissue around the elbow than adults after a serious fracture-dislocation because of their physiologic or pathologic elbow joint laxity.

After the divergent dislocation of the elbow, due to swelling and deformity in the elbow, elbow x-ray films are mostly oblique radiographs, rather than the standard AP and lateral radiographs, so typing is relatively difficult.^[12]^ We recommend the elbow computed tomography scan for associated elbow fracture's patients so as to better understand the fracture types of the children, which also makes it easy to compare between pre- and postreduction and evaluation on therapy effects. The divergent dislocation of the elbow, due to severe soft tissue damage around the elbow, can sometimes be easy for CR. The CR method based on the reverse forces and displacement of injury mechanism is the axial traction supplemented by compressing the proximal radius and ulna. Most of the patients can be successful. Simultaneously, the difficulty degree of CR also indirectly suggests the severity degree of soft tissue injuries around the elbow. Only 2 cases reported in the literature underwent OR. In 1 case, because of the accompanied radial neck fractures, the elbow was instable, and in the other case, because of the soft tissue interposition, the patient underwent open reduction and internal fixation. After the reduction, the stability was determined by using flexion and extension, internal and external rotation of the elbow. In the postoperative elbow x-ray film, the radial longitudinal axis should always point to the capitellum, so as to judge the quality of reduction.

## 4 Conclusion

This case is a 10-year-old girl, who fell, resulting in the divergent elbow dislocation on the right side (transverse type). CR was performed under anesthesia, the dislocation was corrected, but the olecranon fracture showed larger displacement, and also because it was intra-articular fracture, the child underwent the OR and Kirschner's wire internal fixation with addition of a figure 8 wire fixation to stabilize the Olecranon. At the end of the normal follow-up, the active ROM of the right elbow joint was 5° to 130° and with normal rotation.

Therefore, through the treatment of this case and the literature review, we believe that for children, most divergent dislocations of the elbow may achieve a better clinical result with CR, and we also believe that after surgery or CR, in the follow-up, proper function exercise is an important condition for the rehabilitation of children. For such patients, correct diagnosis and timely treatment can help to avoid joint dysfunction of elbow.

## References

[1] Thompson GH Beaty JH Kasser JR Dislocations of the elbow. *Rockwood and Wilkins’ Fractures in Children*. Philadelphia, PA:Lippincott Williams & Wilkins; 2001 1410–1411.

[2] Warmont A Luxation simultane’e du cubitus en dedans et du radius en dehors, complique’e de fracture de l’avant-bras. *Med Chir Prat* 1854; 99:961–963.

[3] DeLee JC Transverse divergent dislocation of the elbow in a child. *J Bone Joint Surg Am* 1981; 63:322–323. 7462287

[4] Carey RP Simultaneous dislocation of the elbow and the proximal radioulnar joint. *J Bone Joint Surg Br* 1984; 66:254–256. 6707063 10.1302/0301-620X.66B2.6707063

[5] Anderson K Mortensen A Gron P Transverse divergent dislocation of the elbow: a report of two cases. *Acta Orthop Scand* 1985; 56:442–443. 4072669 10.3109/17453678508994369

[6] Sovio OM Tredwell SJ Divergent dislocation of the elbow in a child. *J Pediatr Orthop* 1986; 6:96–97. 3941188 10.1097/01241398-198601000-00018

[7] McAullife TB Williams D Transverse divergent dislocation of the elbow. *Injury* 1988; 19:279–280. 3229845 10.1016/0020-1383(88)90046-0

[8] Holbrook JL Green NE Divergent pediatric elbow dislocation. *Clin Orthop Relat Res* 1988; 234:72–74. 3409604

[9] Hemmadi SS Trivedi JM Divergent dislocation of the elbow in a child: a case report. *J Post Grad Med* 1991; 37:221–222. 1841973

[10] Nakano A Tanaka S Hirofuji E Transverse divergent dislocation of the elbow in a six year old boy: a case report. *J Trauma* 1992; 32:118–119. 1732564 10.1097/00005373-199201000-00026

[11] Vincente P Orduna M Transverse divergent dislocation of the elbow in a child: a case report. *Clin Orthop Relat Res* 1993; 294:312–313. 8358935

[12] Shankarappa YK Tello E Ferris BD Transverse divergent dislocation of the elbow with ipsilateral distal radius epiphyseal injury in a seven year old. *Injury* 1998; 29:798–802. 10341909 10.1016/s0020-1383(98)00175-2

[13] Zaricznyj B Transverse divergent dislocation of the elbow. *Clin Orthop Relat Res* 2000; 373:146–152. 10.1097/00003086-200004000-00018 10810472

[14] Altuntas AO Balakumar J Howells RJ Posterior divergent dislocation of the elbow in children and adolescents: a report of three cases and review of the literature. *J Pediatr Orthop* 2005; 25:317–321. 15832146 10.1097/01.bpo.0000153877.20561.8a

[15] Basanagoudar P Pace A Ross D Paediatric transverse divergent dislocation of the elbow. *Acta Orthop Belg* 2006; 72:359–361. 16889152

[16] Nanno M Sawaizumi T Ito H Transverse divergent dislocation of the elbow with ipsilateral distal radius fracture in a child. *J Orthop Trauma* 2007; 21:145–149. 17304072 10.1097/BOT.0b013e318032c4be

[17] Afshar A Divergent dislocation of the elbow in an 11-year-old child. *Arch Iran Med* 2007; 10:413–416. 17604487

[18] Parikh SN1 Lykissas MG Mehlman CT Convergent and divergent dislocation of the pediatric elbow: two case reports and comprehensive review of literature. *J Pediatr Orthop B* 2014; 23:158–167. 24201072 10.1097/01.bpb.0000434242.64440.5a

